# Antimicrobial, Antioxidant, and Cytotoxic Activities of *Ocimum forskolei* and *Teucrium yemense* (Lamiaceae) Essential Oils

**DOI:** 10.3390/medicines4020017

**Published:** 2017-04-01

**Authors:** Nasser A. Awadh Ali, Bhuwan K. Chhetri, Noura S. Dosoky, Khola Shari, Ahmed J. A. Al-Fahad, Ludger Wessjohann, William N. Setzer

**Affiliations:** 1 Department of Pharmacognosy, Faculty of Clinical Pharmacy, Albaha University, P.O. Box 1988, Albaha, Saudi Arabia; 2 Department of Pharmacognosy and Phytochemistry, Faculty of Science, Sana’a University, P.O. Box 13150, Sana’a, Yemen; sharee_8855@yahoo.com; 3 Department of Chemistry, University of Alabama in Huntsville, Huntsville, AL 35899, USA.; bhuwanchhetri89@gmail.com (B.K.C.); nouradosoky@yahoo.com (N.S.D.); 4 Department of Chemistry, Faculty of Science, Albaha University, P.O. Box 2345, Albaha, Saudi Arabia; aalfahad@bu.edu.sa; 5 Leibniz Institute of Plant Biochemistry, Department of Bio-Organic Chemistry, Weinberg 3, 06120 Halle, Germany; wessjohann@ipb-halle.de

**Keywords:** antibacterial, antifungal, free radical scavenging, antineoplastic

## Abstract

**Background:** *Ocimum forskolei* and *Teucrium yemense* (Lamiaceae) are used in traditional medicine in Yemen. **Methods:** The chemical composition, antimicrobial, antioxidant and cytotoxic activities of the essential oils isolated from the leaves of *Ocimum forskolei* Benth. (EOOF) and two different populations of *Teucrium yemense* Deflers., one collected from Dhamar province (EOTY-d), and another collected from Taiz (EOTY-t) were investigated. The antimicrobial activities of the oils were evaluated against several microorganisms with the disc diffusion test or the broth microdilution test. The essential oils were screened for *in-vitro* cytotoxic activity against human tumor cells. EOOF and EOTY-d were screened for free-radical-inhibitory activity using the DPPH radical scavenging assay. **Results:** Sixty-four compounds were identified in (EOOF) representing 100% of the oil content with *endo*-fenchol (31.1%), fenchone (12.2%), τ-cadinol (12.2%), and methyl (*E*)-cinnamate (5.1%) as the major compounds. In EOTY-d, 67 compounds were identified, which made up 91% of the total oil. The most abundant constituents were (*E*)-caryophyllene (11.2%), α-humulene (4.0.%), γ-selinene (5.5%), 7-*epi*-α-selinene (20.1%), and caryophyllene oxide (20.1%), while the major compounds in EOTY-t were α-pinene (6.6%), (*E*)-caryophyllene (19.1%) α-humulene (6.4%), δ-cadinene (6.5%), caryophyllene oxide (4.3%), α-cadinol (9.5%), and shyobunol (4.6%). The most sensitive microorganisms for EOOF were *B. subtilis*, *S. aureus*, and *C. albicans* with inhibition zones of 34, 16, and 24 mm and MIC values of, 4.3 mg/mL, 4.3 mg/mL, and 8.6 mg/mL, respectively. EOTY-t showed antimicrobial activity against *S. aureus*, *B. cereus*, *A. niger*, and *B. cinerea* with MIC values of 0.156, 0.156, 0.313 and 0.313 mg/mL, respectively. Neither essential oil showed remarkable radical inhibition (IC_50_ = 31.55 and 31.41 μL/mL). EOTY-d was active against HT-29 human colorectal adenocarcinoma cell lines with IC_50_ = 43.7 μg/mL. Consistent with this, EOTY-t was active against both MCF-7 and MDA-MB-231 human breast adenocarcinoma cells. **Conclusions:** The antimicrobial activity of *Ocimum forskolei* essential oil against *B. subtilis* and *C. albicans* is consistent with its traditional use in Yemeni traditional medicine to treat skin infections. Both *O. forskolei* and *T. yemense* show wide variations in their respective essential oil compositions; there remains a need to investigate both species botanically, genetically, and phytochemically more comprehensively.

## 1. Introduction

*Ocimum forskolei* Benth. (Lamiaceae) is one of about 65 tropical and subtropical species of *Ocimum*, seven of which are found in Yemen, and these include *O. basilicum* L., *O. tenuiflorum* L., *O. suave* Willd., *O. spicatum* Deflers, *O. gratissimum* L., and *O. forskolei* [1]. Several species of *Ocimum* are aromatic and used as flavoring agents, insect repellents, and traditional medicines [2]. *O. forskolei* ranges in East Africa from Egypt, south to Kenya [3], and the southern Arabian Peninsula, including Yemen [4], Oman [5], and the United Arab Emirates (UAE) [6]. *O. forskolei* is used traditionally in Yemen as a cosmetic, to relieve fever, and to treat skin infections [7]. In UAE, crushed leaves of *O. forskolei* are used to treat headaches, colds, and ear aches, while the juice is used as eye drops or for insect bites [6]. The plant is used in Eritrea as a mosquito repellent and has demonstrated repellent activity [8]. The dichloromethane extract of *O. forskolei* has shown weak antibacterial activity against Gram-positive bacteria, while the methanol extract showed minimal radical-scavenging activity [7]. Previous investigations of *O. forskolei* have shown the essential oil to have weak antioxidant [9] and nematicidal activity [10], and better activities against bacteria and dermatophytes [11].

*Teucrium* is a large genus belonging to the Lamiaceae which contains about 250 species that are widespread over the world, but are most common in Mediterranean climates [2]. *Teucrium* species endemic to Yemen are *T. yemense* Deflers, *T. socotranum* (Balf. f.) Vierh., and *T. balfourii* (Balf. f.) Vierh. Plants from this genus are perennial in the form of herbs, shrubs, and sub-shrubs. *Teucrium* species are widely used in Yemeni folk medicine as antispasmodics and insect repellants [12]. *Teucrium* species are rich in essential oil and are valued as ornamental plants. *Teucrium yemense* is mostly found in Djibouti, Ethiopia, Saudi Arabia, Sudan, and Yemen. It is a perennial aromatic plant possessing sessile oblanceolate leaves and dense terminal heads of white flowers [12]. Essential oil studies of various species of *Teucrium* have shown α-humulene, δ-cadinene, (*E*)-caryophyllene, α-pinene, β-pinene, linalool, 3-octanol, α-cadinol, caryophyllene oxide, 8-cedren-13-ol, and (*E*)-β-farnesene to be the major components of the essential oils. A wide range of biological activity has been reported for *Teucrium* species, some of which are attributed to the high content of essential oil in them. They possess antifungal, antibacterial, larvicidal, antispasmodic, antioxidant, anti-inflammatory, antiulcer, hypoglycemic, antiacetylcholinesterase, and hepatoprotective activities [12].

## 2. Materials and Methods 

### 2.1. Plant Materials

The leaves of *O. forskolei* and *T. yemense* were collected from several mature plants during the flowering stage in May 2010 from Dhamar province, Yemen. The plants were identified by Hassan M. Ibrahim of the Botany Department, Faculty of Sciences, Sana’a University. Voucher specimens (YMP Lam-33 and YMP Lam-36) have been deposited at the Pharmacognosy Department, Sana’a University, Yemen*.* Another sample of *T. yemense* leaves was collected in February 2012 from Taiz town, Yemen. For each sample, the dried leaves (100 g) were hydrodistilled for 3 h in a Clevenger type apparatus according to the European Pharmacopoeia method [13]. The obtained oils were subsequently dried over anhydrous Na_2_SO_4_ and kept at 4 °C until analysis.

### 2.2. Gas Chromatography-Mass Spectrometry (GC-MS)

*O. forskolei* and *T. yemense* essential oils were analyzed by GC-MS using an Agilent 6890 GC with an Agilent 5973 mass selective detector (MSD, operated in the EI mode (electron energy = 70 eV), scan range = 40–400 amu, and scan rate = 3.99 scans/s), and an Agilent ChemStation data system. The GC column was an HP-5ms fused silica capillary with a (5% phenyl)-polymethylsiloxane stationary phase, film thickness of 0.25 μm, a length of 30 m, and an internal diameter of 0.25 mm. The carrier gas was helium with a column head pressure of 48.7 kPa and a flow rate of 1.0 mL/min. Inlet temperature was 200 °C and interface temperature was 280 °C. The GC oven temperature program was used as follows: 40 °C initial temperature, held for 10 min; increased at 3 °C/min to 200 °C; increased 2°/min to 220 °C. A 1% (*w*/*v*) solution of the sample in CH_2_Cl_2_ was prepared and 1 μL was injected using a splitless injection technique. Identification of the oil components was based on their retention indices as determined by reference to a homologous series of *n*-alkanes (C_8_–C_40_), and by comparison of their mass spectral fragmentation patterns with those reported in the literature [14], and stored on the MS library (NIST database (G1036A, revision D.01.00)/ChemStation data system (G1701CA, version C.00.01.080). The percentages of each component are reported as raw percentages based on total ion current without standardization.

### 2.3. Radical Scavenging Assay

For the preliminary test, analytical TLC (thin-layer chromatography) on silica gel plates was developed under appropriate conditions after application of 5 μL of oil solution, dried and sprayed with DPPH solution (0.2%, MeOH). Five minutes later, active compounds appeared as yellow spots against a purple background. Estimation of a radical scavenging effect was carried out by using a DPPH (2,2-diphenyl-1-picrylhydrazyl) free radical scavenger assay in 96-well microtiter plates (MTPs) according to a modified method [15]. A solution of DPPH (Sigma-Aldrich, Munich, Germany) was prepared by dissolving 5 mg DPPH in 2 mL of methanol, and the solution was kept in the dark at 4 °C until use. Stock solutions of the samples were prepared at 2 mg/mL and diluted to different concentrations. Methanolic DPPH solution (5 μL) was added to each well. The plate was shaken for 2 min to ensure thorough mixing before being wrapped in aluminum foil and stored in the dark. A methanolic solution of DPPH served as control. After 30 min, the optical density (OD) of the solution was measured at a wavelength of 517 nm using a microtiter plate ELISA reader (Thermo Scientific, Helsinki, Finland) and the percentage decolorization was calculated as an indication of the antioxidant activity of a sample. Each experiment was carried out in triplicate and IC_50_ (median inhibitory concentration) values were calculated using Origin software (version 7, OriginLab Corp., Northampton, MA, USA). Ascorbic acid (Sigma-Aldrich, Munich, Germany) was used as a positive control.

### 2.4. Antimicrobial Assays

The antimicrobial activities of *O. forskolei* (EOOF) and *T. yemense* (EOTY-d) essential oils were evaluated by the agar disc-diffusion method, as previously described [16]. The microorganisms used were *Escherichia coli* ATCC 10536, *Pseudomonas aeruginosa* ATCC 25619, *Staphylococcus aureus* ATCC 29737, *Bacillus subtilis* ATCC 6633, methicillin-resistant *Staphylococcus aureus* (MRSA), and *Candida albicans* ATCC 2091. Müller-Hinton Agar (MHA) (Merck, Darmstadt, Germany) was used for bacterial culture at 37 °C. Sabouraud dextrose agar (Merck, Darmstadt, Germany) was used to cultivate *C. albicans*.

*T. yemense* (EOTY-t) essential oil was screened for antibacterial activity against the Gram-positive bacteria *Bacillus cereus* (ATCC No. 14579) and *Staphylococcus aureus* (ATCC No. 29213) and the Gram-negative bacteria *Pseudomonas aeruginosa* (ATCC No. 27853) and *Escherichia coli* (ATCC No. 10798). The minimum inhibitory concentrations (MICs) were determined using the microbroth dilution technique [17]. Dilutions of the essential oils were prepared in cation-adjusted Mueller Hinton broth (CAMHB) beginning with 50 µL of 1% *w*/*w* solutions of oils in DMSO plus 50 µL of CAMHB. The oil solutions were serially diluted (1:1) in CAMHB in 96-well plates. Organisms at a concentration of approximately 1.5 × 10^8^ colony-forming units (CFU)/mL were added to each well. Plates were incubated at 37 °C for 24 h, and the final minimum inhibitory concentration (MIC) was determined as the lowest concentration without turbidity. Gentamicin was used as a positive antibiotic control, while DMSO was used as a negative control [17].

The microbroth dilution technique was also applied to determine the antifungal activity of EOTY-t essential oil against *Aspergillus niger* (ATCC No. 16888), *Botrytis cinerea* (ATCC No. 126943), and *Candida albicans* (ATCC No. 90028). For *Aspergillus niger* and *Candida albicans*, dilutions of the essential oils were prepared in YM broth beginning with 50 µL of 1% *w*/*w* solutions of oils in DMSO plus 50 µL of YM broth. The oil solutions were serially diluted (1:1) in YM in 96-well plates. YM broth inoculated with *A. niger* hyphal and *Candida albicans* culture diluted to a McFarland turbidity of 1.0 were added to each well. Plates were incubated at 37 °C for 24 h; amphotericin B was the positive control, while DMSO was used as the negative control. The same process was used for *B. cinerea* as well, except that the inoculum suspension was prepared in potato dextrose broth (PDB) and the inoculum size was adjusted with respective broth medium between 1.0 × 10^6^ and 5.0 × 10^6^ spores/mL by maintaining the optical density (OD) between 0.1 and 0.2 at 625 nm. Dilutions were carried out in the fungal growth medium (malt extract broth). Combination antifungal cyprodinil-fludioxonil was used as the positive control and DMSO was used as the negative control. The 96-well plates were incubated at 25 ± 2 °C for 48 h.

### 2.5. Cytotoxicity Assays

EOOF and EOTY-d were screened for cytotoxicity on HT-29 (human colorectal adenocarcinoma) cells using the XTT assay [18]. HT29 cells were grown in a 5% CO_2_ environment at 37 °C in RPMI 1640 medium without l-glutamine, supplemented with 10% fetal bovine serum, 1% (200 mM) l-alanyl-l-glutamine, and 1.6% HEPES (1 M). Cells were plated into 96-well cell culture plates at 1.5 × 10^3^ cells (HT29) per well. The volume in each well was 100 μL. After 24 h, supernatant fluid was removed by suction and replaced with 100 μL growth medium containing 0.1 μL of a DMSO solution of the essential oil, giving final concentrations of 100, 50, 25, and 12.5 μg/mL for each well. Solutions were added to wells in three replicates. Medium controls without cells and DMSO controls (0.5 μL DMSO/mL) were used. Digitonin (125 µM) was used as a positive control [19]. After the addition of oils, plates were incubated for 72 h at 37 °C in 5% CO_2_; medium was then removed by suction, and 100 μL of fresh medium was added to each well. In order to establish percent growth inhibition rates, the XTT assay for cell viability was carried out according to Cell Proliferation Kit II protocol, (Roche Diagnostics, Mannheim, Germany). After colorimetric readings were recorded (Molecular Devices SpectraMAX M5 microplate reader, 490 nm), average absorbance, growth inhibition, and standard deviations were determined.

EOTY-t was screened for cytotoxic activity against MCF-7 (human breast adenocarcinoma, estrogen receptor positive) and MDA-MB-231 (human breast adenocarcinoma, estrogen receptor negative) cells using MTT-based cytotoxicity assay [20]. Human MCF-7 (ATCC No. HTB-22) and MDA-MB-231 (ATCC No. HTB-26) breast adenocarcinoma cells were grown in a 3% CO_2_ environment at 37 °C in RPMI-1640 medium, supplemented with 10% fetal bovine serum, 100,000 units penicillin, and 10.0 mg streptomycin per liter of medium, 15mM of HEPES, and buffered with 26.7 mM NaHCO_3_, pH 7.35. Cells were plated into 96-well cell culture plates at 2.5 × 10^4^ cells per well. The volume in each well was 100 μL. After 48 h, supernatant fluid was removed by suction and replaced with 100 μL growth medium containing 1.0 μL of DMSO solution of the essential oil (1% *w*/*w* in DMSO), giving a final concentration of 100 μg/mL for each well. Solutions were added to wells in four replicates. Medium controls and DMSO controls (10 μL DMSO/mL) were used. Tingenone was used as a positive control [21]. After the addition of compounds, plates were incubated for 48 h at 37 °C in 5% CO_2_; medium was then removed by suction, and 100 μL of fresh medium was added to each well. In order to establish percent kill rates, the MTT assay for cell viability was carried out [22]. After colorimetric readings were recorded (570 nm, using a SpectraMAX Plus microplate reader, Molecular Devices, Sunnyvale, CA, USA), percent kill was calculated.

## 3. Results and Discussion

### 3.1. Essential Oil Compositions

#### 3.1.1. *Ocimum forskolei*

The chemical composition of the leaf essential oil of *O. forskolei* (EOOF) is listed in Table 1. A total of 64 compounds were identified, accounting for 100% of the essential oil composition. The major components were *endo*-fenchol (31.1%), fenchone (12.2%), τ-cadinol (12.2%), and methyl (*E*)-cinnamate (5.1%). The essential oil composition of *O. forskolei* from Yemen is remarkably different from that reported by Fatope and co-workers from Oman [11]. These workers found the leaf oil to be composed largely of estragole (42%–78%) and linalool (10%–16%), but apparently no fenchone, fenchol, methyl cinnamate, or τ-cadinol. Al-Hajj and co-workers collected the essential oil of *O. forskolei* from Sana’a, Yemen, but their essential oil analysis is incorrect and cannot be compared [9]. Headspace analysis of *O. forskolei* from Eritrea showed (*E*)-β-ocimene, 4-hexen-1-yl acetate, 3-hexenol, 1-octen-3-ol, α-copaene, linalool (major), (*E*)-caryophyllene (major), α-humulene, methyl salicylate, and methyl cinnamate [8]. The large discrepancies in essential oil compositions of *O. forskolei* may reflect taxonomic confusion or hybridization [3,23].

#### 3.1.2. *Teucrium yemense*

The essential oil compositions of two different collections of *T. yemense* are compiled in Table 1. Although there have been several studies on *Teucrium* essential oils, *T. yemense* has not been extensively studied, possibly due to its confined availability. In a previous study, *T. yemense* collected from Taiz (Sabir Mountain, altitude 1300 m) showed a total of 12 identifiable compounds, the major being δ-cadinene (34.9%), (*E*)-caryophyllene (22.7%), α-humulene (6.1%), and α-selinene (5.4%), as well as two unidentified sesquiterpenes (16.5%) [12]. In this current work, two different populations of *T. yemense* were examined, one collected in May 2010 from Dhamar province, and another collected in February, 2012 from Taiz. Although qualitatively similar, there were notable quantitative differences between these two collections and a previous collection from Taiz in 2005 [12]. All three collections showed (*E*)-caryophyllene to be a major component (11.2%–22.7%). δ-Cadinene was the major component from the Taiz sample in 2005 [12], but was only a minor component in the Dhamar sample, while 7-epi-α-selinene (20.1%) and caryophyllene oxide (20.1%) were the major components in the Dhamar sample. α-Humulene was relatively abundant in all three *T. yemense* samples (4.0%–6.4%), while α-cadinol was relatively abundant (2.0 and 9.5%) in the two *T. yemense* samples in this current investigation. Sesquiterpenoids, including (*E*)-caryophyllene, α-humulene, germacrene D, δ-cadinene, caryophyllene oxide, α-cadinol, and τ-cadinol, have been generally abundant in *Teucrium* essential oils (Table 2) [24,25,26,27,28,29,30,31,32,33,34,35,36,37,38,39,40,41,42,43,44,45]. Although relatively abundant in *T. yemense*, 7-*epi*-α-selinene has been found in relatively small amounts in *T. persicum* [46] and *T. capitatum* [33].

### 3.2. Biological Activities

#### 3.2.1. Free Radical Scavenging

*O. forskolei* and *T. yemense* leaf oils from Dhamar (2010) were screened for free-radical-inhibitory activity using the DPPH· radical scavenging assay. Neither essential oil showed remarkable radical inhibition (IC_50_ = 31.55 and 31.41 μL/mL, respectively). This is not surprising; neither essential oil has high concentrations of phenolic compounds such a carvacrol, thymol, or eugenol, which are known to be excellent radical scavenging agents [47]. *endo*-Fenchol, the major component in *O. forskolei* oil, (*E*)-caryophyllene and α-humulene, major components in *T. yemense* oil, have shown only weak antioxidant activities [48].

#### 3.2.2. Antimicrobial Activity

*O. forskolei* and *T. yemense* essential oils were screened for antibacterial and antifungal activity (Table 3). The disc diffusion assay showed *O. forskolei* to have weak antibacterial activity against *Staphylococcus aureus*, but stronger activity against *Bacillus subtilis* with an inhibition zone of 34 mm, and antifungal activity against *Candida albicans* with an inhibition zone of 24 mm. EOTY-d was virtually devoid of antimicrobial activity. Using the broth microdilution assay, EOTY-t showed antibacterial activity against *S. aureus* and *B. cereus* (MIC = 156 μg/mL), as well as antifungal activity against *Aspergillus niger* and *Botrytis cinerea* with MIC of 313 μg/mL.

The antibacterial activity of *O. forskolei* essential oil against *B. subtilis* and *S. aureus* is likely due to methyl (*E*)-cinnamate and τ-cadinol. Methyl cinnamate has shown antibacterial activity against *S. aureus* [49] while τ-cadinol has shown activity against *B. cereus* and *S. aureus* [50]. Apparently neither fenchone nor fenchol are antibacterial [51] and fenchone is also not antifungal [52]. 

The antibacterial activity of EOTY-t essential oil against *S. aureus*, *B. cereus*, *E. coli*, and *A. niger* can be attributed to the relatively high concentrations of (*E*)-caryophyllene, α-humulene, δ-cadinene, and α-cadinol in the oil. (*E*)-Caryophyllene has shown antibacterial activity against each of these organisms [53,54]. α-Humulene has shown antibacterial activity against *B. cereus* and *S. aureus* [53] as well as antifungal activity against *A. niger* [53]. δ-Cadinene was shown to be antibacterial against *Bacillus subtilis* and *Propionibacterium acnes* [55,56], while α-cadinol has shown activity against *B. cereus* and *S. aureus* [50].

#### 3.2.3. Cytotoxic Activity

*O. forskolei* leaf oil was screened for cytotoxic activity against HT-29 human colorectal adenocarcinoma cells, but was inactive (Table 4). EOTY-d was active against this cell line with IC_50_ = 43.7 μg/mL. Consistent with this, EOTY-t was active against both MCF-7 and MDA-MB-231 human breast adenocarcinoma cells.

The cytotoxic activities of *T. yemense* leaf essential oils is likely due to the relatively high concentrations of known cytotoxic components such as (*E*)-caryophyllene (MCF-7 [57], HT-29 [58,59], other cell lines [56]) α-humulene (MCF-7 [57], HT-29 [58,59], other cell lines [60,61]) δ-cadinene [56], caryophyllene oxide (MCF-7 [57], other cell lines [62]), and α-cadinol (MCF-7, HT-29 [63], other cell lines [50]). Furthermore, (*E*)-caryophyllene has been shown to potentiate the cytotoxic activities of other sesquiterpenoids [64]. Interestingly, although *O. forskolei* essential oil was not cytotoxic, one of its major components, τ-cadinol, has shown cytotoxic activity [50]. Fenchone, however, has been shown not to be cytotoxic [57], and there are apparently no reports in the literature about cytotoxicity of *endo*-fenchol.

## 4. Conclusions

In summary, this preliminary phytochemical and bioactivity investigation has described the composition of *Ocimum forskolei* leaf essential oil from Yemen for the first time, and has shown the leaf oil to present moderate antimicrobial properties against *B. subtilis* and *C. albicans*. This activity is consistent with its uses in Yemeni traditional medicine for some skin infections. The variation in the chemical compositions and biological activities of *T. yemense* leaf essential oils can be due to the different environmental factors such as altitude, latitude, or time of collection. Both populations of *T. yemense* showed good cytotoxic activity. There remains the need to follow up this preliminary study and investigate both species more comprehensively in terms of botanical, genetic, phytochemical, and biological properties. This is particularly important because much primary health care in Yemen still relies on traditional herbal practices.

## Figures and Tables

**Table 1:** Leaf essential oil compositions of *Ocimum forskolei* and *Teucrium yemense* from Yemen.

RI_calc_^a^	RI_lit_^b^	Compound	% Composition		
			EOOF	EOTY-d	EOTY-t
936	930	α-Thujene	---	tr ^c^	tr
942	939	α-Pinene	0.5	2.3	6.6
954	954	Camphene	0.3	---	0.1
959	960	Thuja-2,4(10)-diene	---	---	tr
976	975	Sabinene	tr	0.5	tr
979	979	β-Pinene	tr	1.1	3.1
982	979	1-Octen-3-ol	tr	---	---
992	990	Myrcene	0.4	---	0.2
1004	1002	α-Phellandrene	tr	---	---
1009	1005	(3*Z*)-Hexenyl acetate	tr	---	---
1025	1024	*p*-Cymene	0.1	tr	tr
1028	1029	Limonene	2.5	0.5	1.2
1031	1031	1,8-Cineole	0.3	---	---
1067	1070	*cis*-Sabinene hydrate	0.3	---	---
1073	1068	1-Octanol	tr	---	---
1088	1086	Fenchone	12.2	0.3	---
1094	1092	6,7-Epoxymyrcene	0.1	---	---
1100	1096	Linalool	5.7	0.2	0.1
1113	1116	*endo*-Fenchol	31.1	0.1	---
1122	1122	*trans*-Pinene hydrate	0.1	---	---
1125	1127	Chrysanthenone	---	tr	---
1126	1126	α-Campholenal	---	tr	0.1
1137	1140	Nopinone	---	tr	---
1138	1139	*trans*-Pinocarveol	---	0.4	0.2
1141	1141	*cis*-Verbenol	---	tr	0.1
1144	1144	*trans*-Verbenol	---	1.0	0.3
1145	1146	Camphor	6.2	---	---
1157	1159	Sabina ketone	---	tr	---
1162	1164	Pinocarvone	---	---	tr
1165	1169	Borneol	1.0	---	tr
1167	1169	*p*-Mentha-1,5-dien-8-ol	---	---	0.1
1177	1177	Terpinen-4-ol	0.2	0.2	tr
1183	1182	*p*-Methylacetophenone	tr	tr	---
1185	1182	*p*-Cymen-8-ol	0.2	0.1	0.1
1190	1188	α-Terpineol	0.8	0.1	0.1
1195	1195	Myrtenal	---	0.4	0.2
1196	1195	Myrtenol	---	---	tr
1198	1196	Estragole (=Methyl chavicol)	0.2	---	---
1207	1205	Verbenone	---	0.6	0.2
1217	1216	*trans*-Carveol	---	tr	0.1
1219	1220	*endo*-Fenchyl acetate	2.8	---	---
1237	1241	Cuminaldehyde	---	tr	---
1241	1243	Carvone	---	tr	tr
1243	1244	Carvacrol methyl ether	---	---	tr
1252	1252	Geraniol	tr	---	---
1285	1288	Bornyl acetate	0.1	0.4	0.3
1299	1298	*trans*-Pinocarvyl acetate	---	tr	---
1302	1299	Carvacrol	tr	---	0.2
1305	1299	Methyl (*Z*)-cinnamate	0.9	---	---
1324	1326	Myrtenyl acetate	---	1.0	---
1334	1338	δ-Elemene	---	---	0.1
1349	1348	α-Cubebene	0.1	tr	0.1
1375	1376	α-Copaene	0.1	tr	0.6
1385	1378	Methyl (*E*)-cinnamate	5.1	tr	---
1387	1388	β-Bourbonene	---	0.3	1.0
1390	1388	β-Cubebene	0.2	---	0.2
1393	1390	β-Elemene	0.3	0.9	0.3
1406	1403	Methyl eugenol	tr	---	---
1408	1408	(*Z*)-Caryophyllene	---	tr	0.1
1409	1409	α-Gurjunene	---	---	0.2
1416	1412	α-*cis*-Bergamotene	tr	---	---
1419	1419	(*E*)-Caryophyllene	1.1	11.2	19.1
1429	1432	β-Copaene	---	---	0.2
1437	1434	α-*trans*-Bergamotene	3.1	0.1	tr
1439	1439	α-Guaiene	0.4	tr	tr
1444	1441	Aromadendrene	---	---	tr
1454	1454	α-Humulene	0.2	4.0	6.4
1459	1456	(*E*)-β-Farnesene	0.1	0.1	0.1
1461	1454	Alloaromadendrene	---	tr	2.2
1464	1466	*cis*-Muurola-4(14),5-diene	0.5	---	0.1
1467	1467	Ethyl (*E*)-cinnamate	---	tr	---
1474	1479	*trans*-Cadina-1(6),4-diene	---	---	0.2
1476	1477	γ-Gurjunene	---	tr	---
1478	1479	γ-Muurolene	---	---	0.4
1482	1485	Germacrene-D	0.8	0.1	0.4
1484	1479 ^d^	γ-Selinene	---	5.5	0.4
1487	1490	β-Selinene	1.2	0.3	2.5
1495	1494	*epi*-Cubebol	---	---	0.9
1495	1496	Valencene	---	3.7	---
1496	1492	δ-Selinene	0.8	---	---
1497	1500	Bicyclogermacrene	---	---	0.8
1500	1496	Viridiflorene	0.1	---	---
1502	1500	α-Muurolene	---	tr	1.0
1506	1509	Germacrene A	---	---	0.1
1507	1509	α-Bulnesene	0.9	---	---
1510	1505	β-Bisabolene	---	tr	0.1
1516	1513	γ-Cadinene	2.9	---	2.7
1521	1522	7-*epi*-α-Selinene	0.1	20.1	1.3
1523	1522	*trans*-Calamenene	0.2	---	---
1525	1523	δ-Cadinene	0.2	0.4	6.5
1532	1535	10-*epi*-Cubebol	0.1	---	---
1534	1534	*trans*-Cadina-1,4-diene	---	---	0.1
1537	1538	α-Cadinene	tr	---	0.2
1547	1544	*cis*-Sesquisabinene hydrate	---	3.4	0.9
1553	---	Unidentified	---	1.2	---
1557	1561	*cis*-Muurol-5-en-4α-ol	0.1	---	---
1560	1563	(*E*)-Nerolidol	---	tr	---
1565	1565	β-Calacorene	---	tr	---
1566	---	Unidentified	---	0.5	---
1578	1575	Germacrene D-4-ol	---	---	3.1
1584	1583	Caryophyllene oxide	0.2	20.1	4.3
1604	1602	Ledol	---	3.6	0.5
1610	1608	Humulene epoxide II	---	---	0.9
1616	1619	1,10-di-*epi*-Cubenol	1.6	---	0.1
1619	1623	10-*epi*-γ-Eudesmol	---	0.6	0.3
1629	1628	1-*epi*-Cubenol	---	0.6	0.6
1637	1640	Caryophylla-4(12),8(13)-dien-5-ol	---	0.7	0.4
1643	1640	τ-Cadinol	12.2	2.0	5.7
1646	1642	τ-Muurolol	---	0.6	4.9
1647	1646	α-Muurolol (= Torreyol)	---	---	0.7
1648	1649	Methyl (*Z*)-jasmonate	0.2	---	---
1653	1650	β-Eudesmol	0.1	0.9	0.3
1657	1654	α-Cadinol	0.4	2.0	9.5
1660	1663 1666 1661	7-*epi*-α-Eudesmol + Intermediol + *cis*-Calamenen-10-ol	---	3.3	---
1668	1669	*trans*-Calamenen-10-ol	---	0.6	---
1672	1671	*epi*-β-Bisabolol	---	---	0.3
1672	1669	14-Hydroxy-9-*epi*-(*E*)-caryophyllene	---	0.8	---
1674	1676	Cadalene	---	0.4	---
1685	1685	α-Bisabolol	0.1	---	---
1687	1689	*cis*-14-nor-Muurol-5-en-4-one	0.3	---	---
1690	1689	Shyobunol	---	---	4.6
1704	1702	10-Norcalamenen-10-one	---	0.2	---
1738	1740	Oplopanone	---	0.3	tr
1773	1775	*epi*-Cyclocolorenone	---	0.8	---
1803	1806	Nootkatone	---	0.6	---
		Total Identified	100	91.7	98.6

^a^ RI_calc_ = Retention Index calculated with respect to a homologous series of *n*-alkanes on an HP-5ms column; ^b^ RI_lit_ = Retention Index from Adams [14]; ^c^ tr = trace (<0.05%); EOOF = *Ocimum forskolei* Benth.; EOTY-d = *Teucrium yemense* Deflers collected from Dhamar province; EOTY-t = *Teucrium yemense* Deflers collected from Taiz; ^d^ National Institute of Standards and Technology (NIST).

**Table 2:** Major sesquiterpenoid components found in *Teucrium* essential oils.

Major Sesquiterpenoid	*Teucrium* Species	%	Refs.
(*E*)-Caryophyllene	*T. chamaedrys*	47.6	[24]
	*T. polium*	52.0	[24]
	*T. arduini*	35.2	[25]
	*T. turredanum*	15.6–32.6	[26]
	*T. scorodonia* ssp. *baeticum*	33.8	[27]
α-Humulene	*T. alopecurus*	12.3	[28]
	*T. flavum*	8.4	[29]
	*T. marum*	7.2	[30]
	*T. polium*	4.3	[31]
	*T. scorodonia* ssp. *baeticum*	9.1	[27]
	*T. turredanum*	4.7–10.1	[26]
Germacrene D ^a^	*T. sandrasicum*	27.9	[42]
	*T. arduini*	17.0–18.7	[25,43]
	*T. chamaedrys*	16.5–32.1	[24,44,45]
	*T. scorodonia* ssp. *baeticum*	22.2	[27]
δ-Cadinene	*T. montanum*	17.2	[32]
	*T. capitatum*	3.0–9.8	[33]
	*T. maghrebinum*	13.5	[34]
	*T. ramosissimum*	20.0	[35]
	*T. stocksianum*	12.9	[36]
Caryophyllene oxide	*T. orientale* ssp. *taylori*	15.6	[37]
	*T. montbretti*	12.7	[38]
	*T. arduini*	14.6	[39]
α-Cadinol	*T. polium* ssp. *aurasiacum*	46.8	[40]
	*T. polium* ssp. *capitatum*	4.5	[38]
	*T. ramosissimum*	9.9	[35]
	*T. leucocladum*	9.3	[41]
	*T. capitatum*	1.6–9.8	[33]
τ-Cadinol	*T. capitatum*	1.6–24.1	[33]
	*T. montanum*	5.5	[42]
	*T. leucocladum*	5.5	[41]

^a^ Not abundant in *T. yemense* essential oils.

**Table 3:** Antimicrobial activity (minimum inhibitory concentration (MIC), μg/mL) of *Ocimum forskolei* and *Teucrium yemense* leaf essential oils.

Organism	EOOF ^a^	EOTY-t ^b^	Positive Control
*S. aureus*	4300	156	0.61 ^e^
MRSA	8600	nt ^c^	<10.0 ^f^
*B. cereus*	nt	156	1.22 ^e^
*B. subtilis*	4300	nt	<10.0 ^e^
*E. coli*	na ^d^	313	1.22 ^e^
*P. aeruginosa*	na	1250	2.44 ^e^
*C. albicans*	8600	1250	0.61 ^g^
*A. niger*	nt	313	0.61 ^g^
*B. cinerea*	nt	313	<19.5 ^g^

^a^ Antimicrobial activity of *O. forskolei* essential oil determined using the disc diffusion assay; ^b^ Antimicrobial activity determined using the broth dilution assay on *T. yemense* Taiz (2012) essential oil; ^c^ “nt” = not tested; ^d^ “na” = not active (no zone of inhibition); ^e^ Bacterial positive control, gentamicin sulfate; ^f^ MRSA positive control, enoxacin; ^g^ Fungal positive control, amphotericin B.

**Table 4:** Cytotoxic activity (IC_50_, μg/mL) of *Ocimum forskolei* and *Teucrium yemense* leaf essential oils.

Cell Line	EOOF	EOTY-d	EOTY-t
HT-29	na ^a^	43.7 ± 7.1	nt ^b^
MCF-7	nt	nt	24.4 ± 1.8
MDA-MB-231	nt	nt	59.9 ± 4.6

^a^ “na” = not active (0% kill at 100 μg/mL); ^b^ “nt” = not tested.
